# Atrial fibrillation is associated with lean body mass in postmenopausal women

**DOI:** 10.1038/s41598-019-57167-3

**Published:** 2020-01-17

**Authors:** Marie S. Worm, Cecilie L. Bager, Joseph P. M. Blair, Niels H. Secher, Bente J. Riis, Claus Christiansen, Henning B. Nielsen

**Affiliations:** 1 Proscion, Herlev, Denmark; 2 Department of Anaesthesia, Rigshospitalet, University of Copenhagen, Copenhagen, Denmark; 3 Nordic Bioscience, Herlev, Denmark; 4 Sanos Clinic, Herlev, Denmark

**Keywords:** Atrial fibrillation, Risk factors

## Abstract

This study investigated the association between body composition and risk of atrial fibrillation (AF) in postmenopausal women. In a retrospective analysis we assessed data from 5704 postmenopausal women (age 70.7 ± 6.5 yrs.) who in 1999–2001 participated in The Prospective Epidemiological Risk Factor study with body composition assessed by dual-energy X-ray absorptiometry. Outcomes were obtained from Danish Health Registries and body composition association to risk of AF was evaluated by univariable and multivariable Cox Hazard regression. 850 women developed AF after baseline. High lean body mass was associated with increased risk of AF in multivariable analyses, adjusting for body mass index (BMI), height or weight (adjusted for: BMI, hazard ratio (HR) 1.49, 95% Confidence Interval (1.22–1.80); height, HR 1.27 (1.03–1.56); weight, 1.33 (1.06–1.65)). Height and weight were associated with increased risk of AF in multivariable analyses adjusting for body composition measures. When adjusting for total lean mass, only height remained statistically significant (HR 1.34 (1.09–1.64)). In a cohort of elderly Caucasian women, high lean body mass, height and weight were associated with increased risk of AF and the variables remained significant after adjusting for age and other known risk factors of AF.

## Introduction

Obesity is linked to cardiovascular disease (CVD) including atrial fibrillation (AF) and AF is the most common arrhythmia^1^ with prevalence that increases with age. In women, AF presents later in life compared to men and AF seldomly manifests before menopause^2^.

Several risk factors of AF including hypertension, diabetes, heart failure and myocardial infarction have been identified in previous studies^3–5^. While obesity defined by body mass index (BMI; ≥30 kg/m^2^) is a known modifiable risk factor of AF in both men and women^5–7^, height has also been suggested a risk factor for AF^8,9^. Furthermor, other less explored body composition measures may be important for development of AF. In a bio-impedance study including middle aged men and women (approx. 60 years, 47.6% males), lean body mass was associated with increased risk of AF^10,11^. Also, using dual-energy X-ray absorptiometry (DXA) to assess body composition in 60 year old women, lean body mass was associated with increased risk of AF^12^. Furthermore, in older adults total fat mass^9,13^, fat free mass^9^ and height^9^ were associated with risk of AF. Karas *et al*. concluded that their evaluation on 1050 incident AF in 4276 participants is the largest study focused on older adults to report on anthropometric variables, along with measures of fat and fat-free mass^9^. Cohorts on elderly women only are in need as the postmenopausal phase involves high risk for development CVD^14^ concomitant with significant change in body composition. Thus, women loose subcutaneous fat but gain abdominal fat with age^15^ paralleled with a progressive decline in muscle mass and strength and muscle function become impaired^16^. Accumulation of visceral fat involves activation of inflammatory pathways^17^ with potential for development of atrial fibrosis^5^. Also pericardial and epicardial fat accumulation associate to AF^18–20^.

To explore further the AF risk in elderly women data from the Prospective Epidemiological Risk Factor Study (PERF) were applied. The PERF cohort represents a large sample of elderly Caucasian females (n = 5855) who had their body composition assessed by DXA. We aimed to investigate whether different body composition measures (fat and lean body mass) and anthropometric measures (BMI, height and weight) are associated to risk of AF in elderly women.

## Methods

### Study population

The PERF cohort^21^ was established to identify risk factors for age-related diseases and includes Danish post-menopausal women who had either been screened for or participated in clinical prevention trials at the Center for Clinical and Basic Research, Denmark. Among the source population, 8875 women were approached by letter in accordance with approval by the Research Ethics Committee of Copenhagen County, Viborg and Northern Jutland, Denmark (KA 99070 gm). Of these, 732 women had died. Following written informed consent 5855 women (age 70.7 ±  6.5 years) were included in the PERF cohort between 1999–2001^21^ (baseline) giving a response rate of 72% of the women alive at time of enrolment in PERF. Inclusion and exclusion criteria at the time of enrolment was limited to women who were postmenopausal and the study is considered a retrospective analysis of a prospective study-design. All participants attended a physical examination and blood was sampled at baseline. Demographics were obtained through interview by a doctor or a nurse. The interview focused on family history of disease, reproduction and previous health status as well as use of medicine including hormonal replacement therapy (HRT) level of education (elementary, secondary, university), and lifestyle habits such as engagement in physical exercise (hours per week), alcohol consumption (units per week) and smoking habits (never, previous, current). Follow-up was ensured with registry-based data using population-based national registries by matching the unique civil registration number for Danish residents. The cohort is considered to be representative for Danish elderly post-menopausal women^21^ with a detailed description of the PERF cohort published by Neergaard *et al*.^21^.

### Inclusion and exclusion criteria

The present evaluation included all PERF women in whom the diagnosis code of AF in accordance with ICD-8 or ICD-10 was absent at baseline (Table 1). The registries used were the Danish National Patient Register, the Danish National Diabetes Register, and the Danish Register of Causes of Death. Latest linkage used for present study was January 2015.

**Table 1:** Diagnosis and procedure codes.

Disease	ICD-8	ICD-10
**AF**	42793, 42794	I48
**IHD:**		
Diagnosis code	41009–41499	I21, 22, 24, 25
*Procedure code (CABG/PCI)*	*30009, 30019, 30029–30099, 30109–30199, 30240–30245, 30280*	*KFA, KFB, KFC, KFE, KFG, KNK, KFM, KFN, KFP, KFW, KFX*
**PAD**	44020–44029	I70.2, 73.9
**Hypertension**	40009–40499, 41009, 41109, 41209, 41309, 41409	I10–15
**Hyperlipidaemia**	27201, 27900, 27901, 28200	E78.0–78.5
**CHF**	42599, 42709–42719, 42799, 42899	I50
**Thyroid disease**	24200–24509	E00–03, 05, 06, 07.9

AF, atrial fibrillation; IHD, ischemic heart disease; CABG, coronary artery bypass grafting; PCI, percutaneous coronary intervention; PAD, peripheral arterial disease; CHF, congestive heart failure.

### Variables included in analysis

The variables included in the analysis for AF risk were those obtained from the baseline assessment including the DXA derived body compositions. AF was identified as the first registered diagnosis of AF in the Danish National Patient Registry. Also ischaemic heart disease (IHD), congestive heart failure (CHF), peripheral arterial disease (PAD), and thyroid disease prior to or at baseline were identified. Both ICD-8 and ICD-10 diagnostic codes were used (Table 1).

We also included diagnostic variables of hypertension, hyperlipidaemia and diabetes. Hypertension was defined in accordance to ICD-8 or ICD-10 classification or whether a participant was reported to use antihypertensive medication. A participant was also considered with hypertension if the baseline systolic pressure was above 145 mmHg or if the diastolic pressure was higher than 95 mmHg. A 5 mmHg higher limit for hypertension than normally used^22^ was chosen to minimise the risk of false positive. Hyperlipidaemia was defined as (*i*) baseline fasting s-cholesterol above 7.5 mmol/L^23^, (*ii*) relevant ICD-8 or ICD-10 classification, or (*iii*) hyperlipidaemia noted in the baseline questionnaire. A participant was considered diabetic in accordance to the ICD-8 or ICD-10 codes, or if the participant was listed in the National Diabetes register or if the baseline blood sample showed fasting s-glucose ≥7 mmol/L^24^.

### Body composition measures

The included DXA scan derived body composition measures were total body fat mass (total fat mass (kg)), body fat percentage (fat %), total lean body mass (total lean mass (kg)) and lean body mass index (LBMi; total lean mass(kg)/height(m)^2^). The DXA scans were performed on three different machines [Lunar Prodigy (GE Lunar Corporation, Madison, WA), QDR 2000, or QDR 4500 (Hologic Inc., Waltham, MA)] and the body composition measures were calibrated by bone mineral content. The three groups of body composition measures were controlled to be normally distributed and then normalised by the median to assure comparability. Fat % was the percentage fat mass of total mass (total mass = total bone mineral content + total fat mass + lean body mass). In order to compare anthropometric measures and the DXA derived body composition measures weight, height and BMI were included in the analysis. The BMI was categorised as normal (<25 kg/m^2^), overweight (25− < 30 kg/m^2^), or obese (≥30 kg/m^2^). Height, weight and the derived body composition measures were categorised into tertiles: lowest (tertile 1 = T1), middle (tertile 2 = T2) and highest (tertile 3 = T3) with tertile 1 as the reference.

### Statistical analysis

Baseline demographic characteristics of women with and without AF at end of the study were compared by a two-sided t-test for continuous variables and chi-square test for categorical variables. In the event of missing data for one or more variables, subjects were excluded from the individual analysis and the number included in analysis is reported for each individual analysis. The association between AF and the anthropometric measures or DXA scan derived body composition measures were evaluated by univariable proportional Cox hazard regressions. To adjust for confounders, two different multivariable Cox proportional hazards regression analysis were applied. Model 1 adjusted for age (in years) only. Model 2 adjusted for age (in years), previous/current smoker (yes/no), alcohol consumption ≥7 units/week (yes/no), level of education, physical activity ≥1 h/week (yes/no), HRT (yes/no) and comorbidities such as hypertension, hyperlipidaemia, diabetes, IHD, PAD, CHF. The scanner model was added as a covariable due to the data from the DXA scanners were not calibrated for all body composition measures. Models 1 and 2 were performed individually for the 3 anthropometric measures (BMI, height, weight) and the 4 body composition measures obtained from DXA scans (total fat mass, fat %, total lean mass, and LBMi).

To explore the relationship between body composition and the risk of AF, we performed another set of Cox proportional hazards regression analysis using all the variables in model 2 as well as including one anthropometric measure (BMI, height or weight) and one DXA-scan derived body composition measure (lean body mass, LBMi, total fat mass or fat %). Multicollinearity is underreported in epidemiological studies^25^. To test for multicollinearity between the body composition measures and the anthropometric measures, we used the Variance Inflation Factor (VIF)-test^26^. A Cox proportional hazards regression analysis using the variables of model 2 was used for the VIF-test. Since the cut-off value for the VIF-factor has to be interpreted with caution^26^ a cut-off of and above 2.5 was chosen.

R-software (R vers. 3.4.2, R Development Core Team, 2017) was used for analysis. The following packages were used with default settings: rms (V.5.1-3.1) and survival (V.2.4.3-3). Statistical significance was accepted at a *p*-value < 0.05.

## Results

### Baseline characteristics

Of the 5855 women in the PERF cohort, 5704 women (mean age 70.7 ± 6.5 yrs.) had no history of AF at baseline, but 850 women were diagnosed with new-onset AF after baseline (Table 2). Total follow-up was 14.4 years and as 1848 women (404 with AF and 1444 without) died before reaching the end of the follow-up, the average follow-up period was 11.5 years (SD +/− 4.3 years). The average time to AF was 7.6 years (SD +/− 4.1 years).

**Table 2:** Patient Characteristics at baseline.

Cohort Characteristics	Women without AF*, n = 4854		Women with AF*, n = 850		*p*-value
	%	yes/total no.	%	yes/total no.	
**Age at baseline, years**					
Mean ± SD:	69.7 ± 6.5 (n = 4854)		73.0 ± 6.1 (n = 850)		<0.001
Ever Smoking (yes/no)	52.1	2522/4844	55.5	472/850	0.067
Alcohol (≥7 drinks/week)	32.6	1570/4810	32.7	278/849	0.984
**Education**					
Primary (9 yr)	71.2	3448/4841	73.1	621/850	0.507
High school (12 yr)	21.8	1053/4841	20.7	176/850	
University (17 yr)	7.0	340/4841	6.2	53/850	
Exercise weekly (yes/no)	69.4	3359/4842	64.9	552/850	0.011
HRT ever	27.8	1349/4854	29.3	249/850	0.391
**Blood pressure, mmHg**					
Systolic (mean)	149.2 ± 24.0 (n = 4707)		156.0 ± 26.2 (n = 826)		<0.001
Diastolic (mean)	81.7 ± 11.3 (n = 4709)		82.8 ± 12.7 (n = 826)		0.009
Hypertension**	60.1	2918/4854	71.8	610/850	<0.001
Hyperlipidaemia**	17.7	859/4854	14.3	121/850	0.016
Diabetes mellitus**	6.0	291/4854	8.4	71/850	0.012
IHD**	5.3	258/4854	8.6	73/850	<0.001
PAD**	1.3	64/4854	1.4	12/850	0.955
CHF**	0.8	41/4854	2.0	17/850	0.004
Thyroid disease**	2.8	137/4854	4.2	36/850	0.035
**BMI, kg/m**^2^					
Mean ± SD:	26.1 ± 4.2 (n = 4684)		26.6 ± 4.4 (n = 812)		0.002
Normal weight	43.7	2048	40.4	328	0.002
Over-weight	40.2	1881	38.5	313	
Obese	16.1	755	21.1	171	
**Weight, kg**					
Mean ± SD:	67.5 ± 11.6 (n = 4684)		69.1 ± 12.3 (n = 812)		<0.001
T1 (34.0–62.0 kg)	34.3	1605	29.9	243	0.002
T2 (>62, 0 <71.7 kg)	33.2	1553	31.3	254	
T3 (71.7–134.2 kg)	32.6	1526	38.8	315	
**Height, cm**					
Mean ± SD:	160.9 ± 5.9 (n = 4684)		161.2 ± 6.1 (n = 812)		0.185
T1 (134.4–158.4 cm)	34.0	1592	33.0	268	0.098
T2 (>158.4 < 163.4 cm)	33.2	1553	30.4	247	
T3 (163.4–182.9 cm)	32.9	1539	36.6	297	
**DXA: Total fat mass, kg**					
Mean ± SD:	27.9 ± 9.1 (n = 4412)		28.7 ± 9.4 (n = 760)		0.037
T1 (2.3–23.7 kg)	34.0	1498	31.3	238	0.020
T2 (>23.7 < 31.2 kg)	33.6	1482	31.1	236	
T3 (31.2–76.3 kg)	32.5	1432	37.6	286	
**DXA: Fat %**					
Mean ± SD:	43.1 ± 7.9 (n = 4412)		43.5 ± 8.0 (n = 760)		0.275
T1 (7.7–40.4%)	34.0	1502	29.7	226	0.052
T2 (>40.4 < 47.0%)	33.1	1460	34.1	259	
T3 (47.0–64.8%)	32.9	1450	36.2	275	
**DXA: Total lean mass, kg**					
Mean ± SD:	33.5 ± 3.7 (n = 4412)		33.9 ± 3.8 (n = 760)		0.004
T1 (22.5–31.9 kg)	34.3	1512	29.6	225	0.030
T2 (>31.9 < 34.9 kg)	33.2	1465	34.2	260	
T3 (34.9–59.4 kg)	32.5	1435	36.2	275	
**DXA: LBMi, kg/m**^**2**^					
Mean ± SD:	12.9 ± 1.2 (n = 4400)		13.1 ± 1.2 (n = 757)		0.008
T1 (9.1–12.4 kg/m^2^)	34.1	1502	29.9	226	0.067
T2 (>12.4 < 13.4 kg/m^2^)	33.1	1455	34.7	263	
T3 (13.4–22.0 kg/m^2^)	32.8	1443	35.4	268	

*Women diagnosed with AF after baseline and before February 2015.

**Diagnosis registered at the Danish National Patient Registry, identified at baseline examination and/or the National Diabetes register at baseline.

BMI, Body Mass Index; HRT, hormonal replacement therapy; IHD, ischemic heart disease; PAD, peripheral artery disease; CHF, congestive heart failure; fat %, total percentage body fat; LBMi, Lean Body Mass index; T1, tertile 1; T2, tertile 2; T3, tertile 3.

Table 2 shows demographics of women with and without AF. The women with AF after baseline were approx. three years older than women without AF while level of education, alcohol and smoking habits were similar in the two groups. The women with AF exercised less frequently and their systolic and diastolic blood pressures were significantly higher compared to women without AF. In the women with AF hypertension, diabetes, thyroid disease and heart disease including IHD and CHF, were significantly more prevalent than the women without AF. In contrast, hyperlipidaemia was less prevalent in women with compared to the women without AF. Women with AF were significantly taller, weighed more, had a larger total fat mass, larger lean body mass, and larger LBMi compared to the women without AF.

### Body composition and the risk of AF

Table 3 shows the univariable and multivariable Cox hazard regression analysis of the DXA scan derived body composition and anthropometric variables association to risk of AF. The number of participants included in the individual analyses varied between 5119 and 5496.

**Table 3:** Hazard ratios of the risk of AF, univariable and multivariable adjusted.

Factors	Univariable		Model 1*		Model 2**		VIF***
	HR (95% CI); *p*-value	c-stat; [n,cases]	HR (95% CI); *p*-value	c-stat; [n,cases]	HR (95% CI); *p*-value	c-stat; [n,cases]	
**BMI, kg/m**^2^							
normal weight	ref	0.52; [5496, 812]	ref	0.67; [5496, 812]	ref	0.69; [5124, 757]	ref
overweight	1.00 (0.86–1.17); 0.996		1.01 (0.87–1.18); 0.870		0.99 (0.84–1.16); 0.898		3.61
obese	1.33 (1.10–1.60); 0.003		1.44 (1.20–1.74); <0.001		1.27 (1.03–1.55); 0.023		5.28
**Weight, kg**							
T1	ref	0.53; [5496, 812]	ref	0.68; [5496, 812]	ref	0.69; [5124, 757]	ref
T2	1.01 (0.85–1.21); 0.872		1.12 (0.94–1.34); 0.194		1.13 (0.94–1.35); 0.204		3.45
T3	1.24 (1.05–1.46); 0.012		1.54 (1.30–1.83); <0.001		1.44 (1.21–1.73); <0.001		8.92
**Height, m**							
T1	ref	0.52; [5496, 812]	ref	0.68; [5496, 812]	ref	0.69; [5124, 757]	ref
T2	0.89 (0.75–1.06); 0.188		1.09 (0.92–1.30); 0.315		1.07 (0.89–1.29); 0.454		1.82
T3	1.05 (0.89–1.24); 0.538		1.52 (1.28–1.81); <0.001		1.53 (1.28–1.83); <0.001		3.05
**Total fat mass, kg**							
T1	ref	0.53; [5172, 760]	ref	0.67; [5172, 760]	ref	0.69; [5134, 760]	ref
T2	0.95 (0.79–1.14); 0.570		0.99 (0.83–1.19); 0.950		1.00 (0.83–1.20); 0.994		5.03
T3	1.17 (0.98–1.39); 0.078		1.30 (1.09–1.54); 0.003		1.23 (1.03–1.47); 0.021		3.05
**Fat %**							
T1	ref	0.52; [5172, 760]	ref	0.67; [5172, 760]	ref	0.69; [5134, 760]	ref
T2	1.11 (0.93–1.33); 0.245		1.12 (0.93–1.33); 0.228		1.12 (0.94–1.34); 0.210		3.59
T3	1.17 (0.99–1.40); 0.073		1.23 (1.03–1.47); 0.022		1.16 (0.97–1.39); 0.107		7.24
**Total lean mass, kg**							
T1	ref	0.52; [5172, 760]	ref	0.68; [5172, 760]	ref	0.69; [5134, 760]	ref
T2	1.13 (0.94–1.35); 0.185		1.26 (1.06–1.51); 0.011		1.26 (1.05–1.51); 0.011		2.45
T3	1.19 (1.00–1.43); 0.048		1.55 (1.30–1.85); <0.001		1.51 (1.26–1.81); <0.001		5.19
**LBMi, kg/m**^**2**^							
T1	ref	0.52; [5157, 757]	ref	0.67; [5157, 757]	ref	0.69; [5119, 757]	ref
T2	1.18 (0.99–1.41); 0.066		1.16 (0.97–1.39); 0.100		1.15 (0.96–1.37); 0.135		2.04
T3	1.20 (1.01–1.44); 0.042		1.21 (1.01–1.44); 0.037		1.15 (0.96–1.38); 0.130		4.03

The anthropometric (BMI, height and weight) and DXA scan derived body composition measures (Total fat mass, fat %, total lean mass and LBMi) have been analysed individually, not adjusted for each other.

*Variables adjusted for: age.

**Variables adjusted for: age, alcohol, smoking, education, exercise, HRT, hypertension, hyperlipidaemia, diabetes, IHD, PAD, CHF, thyroid disease and scanner model.

***With all variables of model 2, all anthropometric and body composition measures. All variables adjusted for and had a VIF < 2.5.

HR, Hazard ratios; 95% CI, 95% confidence intervals; c-stat, c-statistics; ref, reference; fat %, total percentage of body fat; LBMi, Lean Body Mass index.

Obese BMI, weight and height in T3 were significantly associated with an increased risk of AF in the two multivariable models. In the univariable analysis only obese BMI was significantly associated with increased risk of AF. Of the DXA scan derived body composition measures total fat mass and fat % in T3 were significantly associated with increased risk of AF in model 1 that adjusted for age only. The measures for lean mass, total lean mass and LBMi, total lean mass and LBMi in T3 were significantly associated with increased risk of AF in the univariable analysis. In the multivariable model 1 and 2, the T2 and T3 of total lean mass were significantly associated with increased risk of AF. The T3 of LBMi was associated with increased risk of AF in model 1. C-stat of the univariable analyses reached 0.52 or 0.53.

### Multicollinearity

Multicollinearity was noted for all anthropometric variables and DXA scan derived measures, except for the T2 data on height (VIF 1.82), total lean mass (VIF 2.45) and LBMi (VIF 2.04) T2 (Table 3). Due to this multicollinearity, multivariable Cox hazard regression adjusting the DXA scan derived body composition measures for the anthropometric measures and vice versa were conducted. Table 4 shows that only total lean mass remained significantly associated with an increased risk of AF when adjusting for height, weight or BMI, but failed to remain significant when adjusting for both height and weight. Weight in T3 was significantly associated with increased risk of AF when adjusting for fat %, total fat mass and LBMi, but when adjusting for lean mass the association failed to remain significant. In all four analyses height in T3 was associated with a 34–56% increased risk of AF when adjusting for body fat, total fat mass, total lean mass, and LBMi.

**Table 4:** Multivariable adjusted hazard ratios of the risk of AF, adjusted for anthropometric or body composition measures.

Factors	BMI	Height	Weight	Height + Weight
	HR (95% CI); *p*-value	HR (95% CI); *p*-value	HR (95% CI); *p*-value	HR (95% CI); *p*-value
**Total fat mass, kg**				
T1	ref	ref	ref	ref
T2	0.99 (0.78–1.22); 0.891	0.98 (0.82–1.17); 0.812	0.82 (0.67–1.01); 0.065	0.87 (0.70–1.07); 0.180
T3	1.19 (0.89–1.59); 0.238	1.15 (0.96–1.38); 0.122	0.78 (0.58–1.05); 0.102	0.87 (0.64–1.18); 0.379
**Fat %**				
T1	ref	ref	ref	ref
T2	1.06 (0.87–1.29); 0.561	1.13 (0.94–1.35); 0.191	0.97 (0.80–1.17); 0.722	1.04 (0.85–1.26); 0.713
T3	1.02 (0.79–1.33); 0.842	1.18 (0.99–1.42); 0.071	0.84 (0.66–1.06); 0.145	0.99 (0.76–1.27); 0.915
**Total lean mass, kg**				
T1	ref	ref	ref	ref
T2	1.25 (1.04–1.50); 0.015	1.14 (0.94–1.37); 0.186	1.19 (0.99–1.44); 0.064	1.09 (0.90–1.33); 0.379
T3	1.49 (1.22–1.80); <0.001	1.27 (1.03–1.56); 0.024	1.33 (1.06–1.65); 0.012	1.15 (0.90–1.45); 0.258
**LBMi, kg/m**^**2**^				
T1	—	ref	ref	ref
T2	—	1.19 (0.99–1.42); 0.062	1.08 (0.90–1.29); 0.431	1.14 (0.95–1.37); 0.162
T3	—	1.21 (1.01–1.46); 0.036	0.97 (0.80–1.18); 0.764	1.10 (0.89–1.35); 0.367
Factors	Fat %	Total fat mass	Total lean mass	LBMi
	HR (95% CI); *p*-value	HR (95% CI); *p*-value	HR (95% CI); *p*-value	HR (95% CI); *p*-value
**BMI, kg/m**^**2**^				
normal weight	ref	ref	ref	—
overweight	0.93 (0.76–1.13); 0.460	0.85 (0.67–1.08); 0.179	0.92 (0.78–1.09); 0.326	—
obese	1.19 (0.90–1.56); 0.216	0.98 (0.72–1.35); 0.918	1.08 (0.87–1.35); 0.466	—
**Weight, kg**				
T1	ref	ref	ref	ref
T2	1.16 (0.95–1.43); 0.146	1.34 (1.04–1.72); 0.021	1.04 (0.86–1.26); 0.658	1.12 (0.93–1.35); 0.226
T3	1.57 (1.24–1.99); <0.001	1.87 (1.34–2.62); <0.001	1.23 (1.00–1.52); 0.055	1.45 (1.19–1.76); <0.001
**Height, m**				
T1	ref	ref	ref	ref
T2	1.08 (0.90–1.29); 0.422	1.06 (0.88–1.27); 0.549	0.99 (0.81–1.19); 0.888	1.08 (0.90–1.30); 0.410
T3	1.53 (1.28–1.83); <0.001	1.49 (1.24–1.79); <0.001	1.34 (1.09–1.64); 0.005	1.56 (1.31–1.87); <0.001

n, cases: 5119, 757, c-statistics for all analyses between 0.688–0.695.

All analyses adjusted for age, alcohol, smoking, education, exercise, HRT, hypertension, hyperlipidaemia, diabetes, IHD, PAD, CHF, thyroid disease and scanner model.

HR, Hazard ratios; 95% CI, 95% confidence intervals; c-stat, c-statistics; ref, reference; fat %, total percentage of body fat; LBMi, Lean Body Mass index.

## Discussion

In this study on elderly post-menopausal women who were followed for 11.5 years, high lean body mass, high total fat mass, obese BMI, height and weight were all associated with an increased risk of atrial fibrillation (AF). Importantly, accumulation of fatty tissue to obese body stature was associated with AF risk in both univariable and multivariable analysis supporting AF risk evaluations in mixed gender populations^7,9,13,27^. When testing for correlations between the anthropometric measures and DXA scan derived body composition measures, lean body mass, weight and height remained associated with increased risk of AF.

The risk of developing AF doubles every 10^th^ years of age^28^ and in a number of studies evaluating anthropometric measures such as BMI, obesity was associated with risk of AF^7,9,13,27^. The importance of height for AF risk in the elderly (age 72.4 years) was indicated by analysis of data from participants in the Cardiovascular Health Study^8,9^. Our data failed to yield a univariate association between height and risk of AF but when adjusting for age and potential confounders, height was associated with increased risk of AF. Thus, attempts to avoid stratification for age could mask the association between height and risk of AF, especially in elderly people. The c-statistics support that analysis should be adjusted for age.

In women, AF presents differently than in men^2^. Women are older at AF onset and they experience worse and longer-lasting symptoms^2^ with a higher risk of severe outcome including stroke^5,29^. These sex-related differences in manifestation of AF are not understood^5^. Men are taller than women, and also body composition could differentiate sex-related manifestation of AF. Thus, height of a person and atrial enlargement are risk factors of AF^30^. In the Framingham study and in the Framingham Offspring cohort, the risk of AF related to obesity diminished when adjusting for left atrial diameter^7^. Structural remodelling including atrial wall fibrosis^31^ mediates AF and although the atria are smaller in women, tendency to develop atrial wall fibrosis is greater in women than in men^5^ and obesity-induced inflammation may lead to atrial fibrosis and AF^32,33^. Notably, pericardial adipose tissue and in turn adipocyte infiltration causes fibrosis^32,33^ that disturbs cardiac electrical conduction^32^.

In a mixed gender cohort (average age range approximately 60 years,)^10,11^, larger weight, height, BMI, waist circumference, bioimpedance derived measures of body fat mass, fat % and lean body mass were associated with increased risk of AF^11^. Lean body mass was associated with an increased risk of AF when adjusting for any of the other anthropometric measures (BMI, height, weight, fat mass, fat %, hip circumference, waist circumference, and waist/hip-ratio)^10^. When adjusting the other measures for lean body mass, only height was associated with increased risk of AF^10,11^. Also, in a multiracial cohort an association between DXA-scan derived measures of lean body mass and risk of AF^12^ is described and a mendelian randomization study points to the importance of lean body mass and fat mass^34^.

Multicollinearity seems underreported in epidemiological studies^25^ and here evaluated by the VIF test^26^, although the cut off values of the VIF-test remain debated^25^. As expected, multicollinearity was identified and lean body mass is not considered the single driver of associations between body composition and increased risk of AF. The relationship between AF and other measures of body composition and anthropometry needs to be considered.

### Clinical relevance

The clinical relevance is that large body stature might mediate AF^35^. The modifiable risk factors of AF are important when trying to decrease risk of developing AF. Height is a fixed value, but weight and obesity are modifiable. Getting access to data on a patients’ lean mass requires, e.g. bioimpedance or DXA scans, whereas assessment of weight and height are easier on-the-spot measures. Of importance, the obese person needs a large skeletal muscle mass to carry the fat mass. Cardiac hypertrophy may develop secondary to increased cardiac output and may further increase risk of AF^35^. Yet, how obesity mediates AF needs to be established.

### Strength and limitations

The study is restricted to elderly females of Caucasian origin. Information regarding life-style habits and use of medication was obtained through interview, and thus based on memory with potential risk for type 2 error and recall bias. We consider this risk of minimal importance since the questionnaire was performed by professional health-care staff and the questions were simple. It is also a limitation that only four women had a BMI <18.5 kg/m^2^.

A strength of the study is the prospective design and the long follow-up period. Likewise, the overall good quality of variables for analysis including DXA scans and access to the Danish registries add to the strength. Secondly, when differentiation between occurrence of atrial fibrillation *vs*. atrial flutter is avoided, the AF diagnosis is highly valid^36^. Although use of diagnosis in registries may have limitations, the national Danish registries are unique in that they are the oldest where individuals are coded in accordance to their personal identification number. Further, data were cross-referenced with other national databases. In addition, completeness of the different diagnoses including CVD is high in Danish registries^37,38^ except that uncomplicated hypertension and diabetes may be incomplete. Adding information about previous hypertensive treatment, blood pressure at baseline and a diagnosis of hypertension improved quality of the diagnosis of hypertension in our study. Regarding diagnosis of diabetes we added information from the Danish National Diabetes Register and blood glucose levels indicative of diabetes.

In conclusion, in a cohort of elderly Caucasian women who were followed for 11.5 years, high lean body mass, height and weight were associated with increased risk of AF. This effect remained significant after adjusting for age and other known risk factors of AF. We suggest that tall elderly women with large muscle mass need specific attention on AF risk.

## Data Availability

The data are not publicly available due to legal and ethical reasons. The data are however available on request from the corresponding author on the condition that the researchers have the appropriate permissions from the Danish Data Protection agency and sign a confidentiality agreement.
